# Nutritional state influences Nociceptin/Orphanin FQ peptide receptor expression in the dorsal raphe nucleus

**DOI:** 10.1016/j.bbr.2009.09.017

**Published:** 2009-09-16

**Authors:** Magdalena J. Przydzial, Alastair S. Garfield, Daniel D. Lam, Stephen P. Moore, Mark L. Evans, Lora K. Heisler

**Affiliations:** 1 Department of Pharmacology, University of Cambridge, Cambridge, CB2 1PD, UK; 2 Metabolic Research Laboratories, Institute of Metabolic Science, Addenbrooke’s Hospital, University of Cambridge, Cambridge CB2 2QQ, UK

**Keywords:** Nociceptin/orphanin FQ (N/OFQ), nociceptin/orphanin FQ peptide (NOP) receptor, dorsal raphe nucleus (DRN), hypothalamus, expression

## Abstract

Agonists of the nociceptin/orphanin FQ (N/OFQ) peptide (NOP) receptor stimulate food intake. Concordantly, neuroanatomical localization of NOP receptor mRNA has revealed it to be highly expressed in brain regions associated with the regulation of energy balance. However, the specific mechanisms and neurochemical pathways through which physiological N/OFQ influences appetite are not well understood. To investigate this, we examined nutritional state associated changes in NOP receptor mRNA levels throughout the rostrocaudal extent of the rat brain using *in situ* hybridization histochemistry (ISHH) and quantitative densitometry analysis. We observed a significant downregulation of NOP receptor mRNA in the dorsal raphe nucleus (DRN) of fasted rats compared to free-feeding rats. In contrast, no difference in NOP receptor mRNA expression was observed in the supraoptic, parventricular, ventromedial, arcuate or dorsomedial nuclei of the hypothalamus, the red nucleus, the locus coeruleus or the hypoglossal nucleus in the fasted or fed state. These data suggest that the endogenous N/OFQ system is responsive to changes in energy balance and that NOP receptors specifically within the DRN may be physiologically relevant to N/OFQ’s effects on appetite.

Nociceptin/orphanin FQ (N/OFQ) is the endogenous ligand of the G_i_ protein-coupled receptor, N/OFQ peptide (NOP) (also referred to as opioid receptor-like 1 (ORL-1) or opioid receptor-4 (OP4)) [1–3]. Central administration of exogenous N/OFQ increases food intake [4–12]; an effect attenuated by pretreatment with NOP receptor antagonists [7, 10] or antisense oligodeoxynucleotides directed against the NOP receptor [6]. Moreover, the appetite stimulating effects of N/OFQ are not observed in NOP receptor deficient mice [10].

Although the mechanisms of N/OFQ mediated feeding remain to be determined, it has been proposed that it is through the suppression of anorectic signaling, rather that the activation of orexigenic pathways, that N/OFQ elicits its appetitive effects; a notion consistent with the inhibitory nature of the activated G_i_-coupled NOP receptor [4–12]. This hypothesis is supported by the findings that fasting *reduces* N/OFQ and NOP receptor gene expression in specific brain nuclei and that central N/OFQ administration has no additive effect on feeding when given to food-deprived rats [11].

The neuroanatomical distribution of the NOP receptor, which is expressed within a number of regions associated with the modulation of appetite, lends further credence to the physiological effects of N/OFQ [13, 14]. More specifically, NOP receptor mRNA is detectable within the arcuate nucleus (ARC), paraventricular nucleus (PVH) dorsomedial nucleus (DMH) and ventromedial nucleus (VMN) of the hypothalamus, nuclei with an established involvement in influencing hunger and/or satiety [15, 16]. NOP receptors are also expressed in the DRN, a primary source of forebrain serotonin, a neurotransmitter which plays an important role in the modulation of feeding behavior (reviewed in [17, 18]). Here we investigate the effects of nutritional state on NOP receptor expression at these and other sites highly expressing this receptor, such as the supraoptic nucleus (SO), the red nucleus (RN), the locus coeruleus (LC) and hypoglossal nucleus (12N).

Adult male Sprague-Dawley rats weighing 280–300 g (Charles River) were individually housed with water and rat chow pellets (Eurodent Diet, PMI Nutrition International) available *ad libitum* in a light-controlled (12 h on/12 h off) and temperature-controlled (21.5°C to 22.5°C) environment, except where noted otherwise. All procedures used were in accordance with the guidelines for the care and use of animals established by the UK Animals (Scientific Procedures) Act 1986. Fasted rats had food removed from their cages for 24 h prior to perfusion, while fed rats had continuous free access to pelleted food and water (n = 5–6/group).

Brain tissue was collected during the light cycle. Rats were deeply anesthetized with pentobarbitone (50 mg/kg of body weight, i.p.) and perfused transcardially with diethylpyrocarbonate (DEPC)-treated phosphate buffered saline (PBS) followed by 10% neutral buffered formalin (Sigma). Brains were removed, post-fixed in 10% formalin for 4 h at 4°C and then submerged for 18–36 h in 20% sucrose in DEPC-treated PBS at 4°C. The processed brains were sectioned coronally on a freezing sliding microtome at 30 µm and collected in 5 equal series of adjacent tissue. One series was thaw-mounted onto SuperFrost slides (Fisher Scientific), air dried, and stored in a desiccated box at −20°C.

Expression of NOP receptor mRNA was detected by *in situ* hybridization histochemistry (ISHH). The NOP receptor riboprobe was synthesized by PCR using cDNA obtained from normal rat brain. A 290 bp fragment, corresponding to nucleotides 1065–1355 of the NOP receptor gene (accession no. NM_031569), was amplified using primers specific to the rat gene sequence (F - 5’ CTG AAT TCA TGA GAA CTT CAA GGC CTG C-3’ and R - 5’AGA AGC TTA TCC TGA TCC AAA AGA AAA GC-3’). The probe fragment was cloned into pcDNA 3.1 plasmid (Invitrogen) and successful insertion verified by DNA sequencing (Geneservice, UK). The recombinant plasmid was linearized by restriction digest and subjected to *in vitro* transcription with a T7 RNA polymerase (antisense) or SP6 RNA polymerase (sense) in the presence of ^35^S-labeled UTP, according to the manufacturer’s instructions (Ambion). The [^35^S]-NOP receptor riboprobes were then diluted to 2×10^7^ cpm/ml in a hybridization solution composed of 50% formamide, 20mM Tris-HCl pH 7.5, 0.02% sheared ssDNA (Sigma), 0.1% total yeast RNA (Sigma), 0.01% yeast tRNA (Gibco), 20% dextran sulfate, 0.3M NaCl, 2mM EDTA pH 8.0, Denhardt’s solution (Sigma), 100mM DTT, 0.2% SDS, and 0.2% sodium thiosulfate (Sigma).

The protocol for ISHH was modified from that previously reported [19, 20]. Before hybridization, brain sections were fixed in 4% formaldehyde in DEPC-treated PBS for 20 min at 4°C, dehydrated in ascending concentrations of ethanol, cleared in xylene for 15 min, rehydrated in descending concentrations of ethanol, and placed in prewarmed sodium citrate buffer (95–100°C, pH 6.0). Slides in sodium citrate buffer were then placed in an LG Intellowave commercial microwave oven for 10 min at 20% power (95–100°C) before being dehydrated in ascending concentrations of ethanol, and air-dried. Hybridization solution (containing radiolabeled riboprobe) and a coverslip were applied to each slide, and sections were incubated for 12–16 h at 57°C. After this time the coverslips were removed, and slides were washed with 2× sodium chloride/sodium citrate buffer (SSC). Sections were then incubated in 0.002% RNase A (Qiagen) for 30 min, followed by sequential washes in decreasing concentrations of SSC. The sections were dehydrated in ascending concentrations of ethanol with 0.3 M ammonium acetate (NH_4_OAc) followed by 100% ethanol. Slides were air-dried and placed in X-ray film cassettes with BMR-2 film (Kodak) for 72 h. Finally, the films were developed on an OPTIMAX X-ray film processor (PROTEC).

For quantification of NOP receptor mRNA levels, autoradiographic images of [^35^S]-labeled brain sections were analyzed with digital analysis software, ImageJ (National Institutes of Health, USA). Analysis of NOP receptor expression was performed at multiple levels from nine brain regions: The SO (−0.84, −0.96, −1.08, −1.20, −1.32, −1.44 mm from bregma), PVH (−1.32, −1.44, −1.56, −1.72, −1.80, −1.92 mm from bregma), VMN (−2.40, −2.52, −2.64, −2.76, −2.92, −3.00 mm from bregma), ARC (−2.40, −2.52, −2.64, −2.76, −2.92, −3.00 mm from bregma), the DMH (−3.12 and −3.24 mm from bregma), RN (−5.88, −6.00, −6.12, −6.24, −6.36, −6.48 mm from bregma), DRN (−7.56, −7.68, −7.80, −7.92, −8.04, −8.16 mm from bregma), LC (−9.48, −9.60, −9.72, −9.84, −9.96 mm from bregma) and 12N (−13.56, −13.68, −13.80, −13.92, −14.04, −14.16 mm from bregma). The [^35^S]-labeled NOP receptor signal density (minus background) was determined by assessing the mean of the optical density of each area of interest. In the case of bilateral nuclei, an average optical density was calculated from both sides of the brain. To account for potential variation in background density between films, the optical density of each region within a brain was normalized to the mean optical density of the cerebral cortex (−2.40, −2.52, −2.64, −2.76, −2.92, −3.00 mm from bregma) of that brain. Any differences detected in NOP receptor expression within a specific brain region were confirmed by a researcher blind to experimental conditions. Nuclei were identified in accordance with *The Rat Brain in Stereotaxic Coordinates* (5^th^ Ed) by Paxinos and Watson [21]. Data are expressed as mean ± S.E.M. Independent sample *t*-tests were used to analyze data comparing NOP receptor expression in fed and fasted rats in each brain region. For all analyses, significance was assigned at the *p*<0.05 level. Sense probe analysis failed to reveal a detectable hybridization signal, confirming the specificity of the antisense NOP receptor riboprobe (Fig 1).

Optical density analysis revealed that 24 hour food deprivation produced a significant decrease in NOP receptor mRNA in the DRN (t(9) = 2.01, *p*<0.05; Fig. 1, Fig. 2), a nucleus expressing the anorectic neurotransmitter serotonin (reviewed in [17, 18]). Expression of the NOP receptor in this region is consistent with immunohistochemical studies which reveal a dense network of N/OFQ-immunoreactive terminals [22]. Moreover, 5,7-dihydroxytryptamine mediated ablation of DRN serotonergic neurons reduces DRN N/OFQ binding and suggests that NOP receptors are expressed on serotonin neurons and thus may be capable of directly modulating neuronal activity [23]. Complementing these studies, NOP receptor agonist administration into the DRN of freely behaving rats decreases serotonin efflux, an effect also observed in N/OFQ treated DRN slices in vitro [24, 25]. The observation that DRN NOP receptor mRNA is significantly reduced in response to food deprivation provides support for a physiological role for endogenous DRN NOP receptors in nutritional state. The modulation of anorectic serotonergic signaling by the NOP receptor is consistent with the currently proposed model of N/OFQ orexigenesis; acting by inhibiting anorectic rather than activating orexigenic pathways.

In contrast to the DRN, no observable changes in NOP receptor mRNA was observed within the canonical satiety centers of the hypothalamus, the VMN or ARC (Fig. 1, Fig. 2) [15, 16]. These findings are consistent with those of Rodi *et al.* and suggest that expression of NOP receptors in the ARC and VMN are not responsive to changes in energy availability/appetite [11]. However, we did not detect a fasting associated decrease in NOP receptor expression within the PVH (Fig. 1, Fig. 2), as reported by Rodi *et al.* [11]. This disparity may be explained by way of methodological differences. Specifically, whilst the fasted animals used by Rodi *et al* experienced food deprivation conditions prior to a 16 hour fast, those used in the present study were naive to food deprivation and fasted for 24 hours. Furthermore, Rodi *et al* reported the optical density of NOP receptor at two levels of the PVH, whereas we assessed expression across six neuroanatomical levels. Thus, it is possible that regional and/or temporal variation in NOP receptor expression and regulation within the PVH could underlie this seemingly discordant observation. In addition to the PVH, Rodi *et al.* also reported a significant reduction in NOP receptor mRNA in response to fasting in the central nucleus of the amygdala and a more modest reduction in the lateral hypothalamus [11].

Here we examined whether NOP receptor mRNA is altered in response to nutritional state in two other hypothalamic sites, the DMH and SO. Neurons within the DMH express leptin receptors and are leptin responsive; leptin is key anorectic hormone released from adipose tissue [26]. Given that NOP receptors are also highly expressed in this brain region, we investigated whether DMH NOP receptor mRNA is altered by the fed or fasted state. No changes were observed (Fig. 1; Fig 2). The presence of anorectic oxytocin and vasopressin expressing neurons [27], in addition to innervation from ARC POMC neurons [28], has highlighted the potential involvement of the SO in appetite. Furthermore, expression of the G_i_-coupled NOP receptor within this region and the decrease in c-Fos immunoreactivty observed upon N/OFQ administration, suggest that the SO may be a direct target for the orexigenic effects of N/OFQ [29]. However, no changes in NOP receptor mRNA within the SO in response to food deprivation were found (Fig. 1, Fig. 2).

We also assessed NOP receptor expression within two regions associated with enabling the mechanical act of food consumption, the RN and 12N [30, 31]. These regions did not exhibit differential NOP expression in the fed or fasted state (Fig. 1, Fig. 2). Lastly, as a control, we examined the LC, a noradrenergic nucleus heavily innervated by the hypothalamus but not classically associated with food intake. Again, no differences in NOP receptor expression were observed (Fig. 1, Fig. 2).

In conclusion, ingestive behavior is a highly regulated process that is modulated by numerous interacting central pathways. N/OFQ is a recently identified neuropeptide that influences appetite through its receptor, NOP. NOP receptor mRNA is expressed in a number of regions influencing food intake [13, 14] and the activity of neurons in many of these areas is affected by N/OFQ or NOP receptor-selective synthetic ligands [23, 24, 32, 33]. To further elucidate specific nuclei responsive to N/OFQ and appetite, we analyzed nutritional state-associated NOP receptor expression in multiple brain regions associated with ingestive behavior. Here we report that NOP receptor expression is influenced by acute changes in nutritional state, indicating that hunger/satiety may dynamically influence its expression. Moreover, we observed that this effect is site specific. We demonstrate that NOP receptor levels in the SO, PVH, VMN, ARC, DMH, RN, LC and 12N are unaffected by food deprivation. In contrast, nutritional state alters the expression of the NOP receptor within the DRN, suggesting that this population of NOP receptors may be specifically physiologically relevant to N/OFQ modulated appetite.

## Figures and Tables

**Fig. 1:**
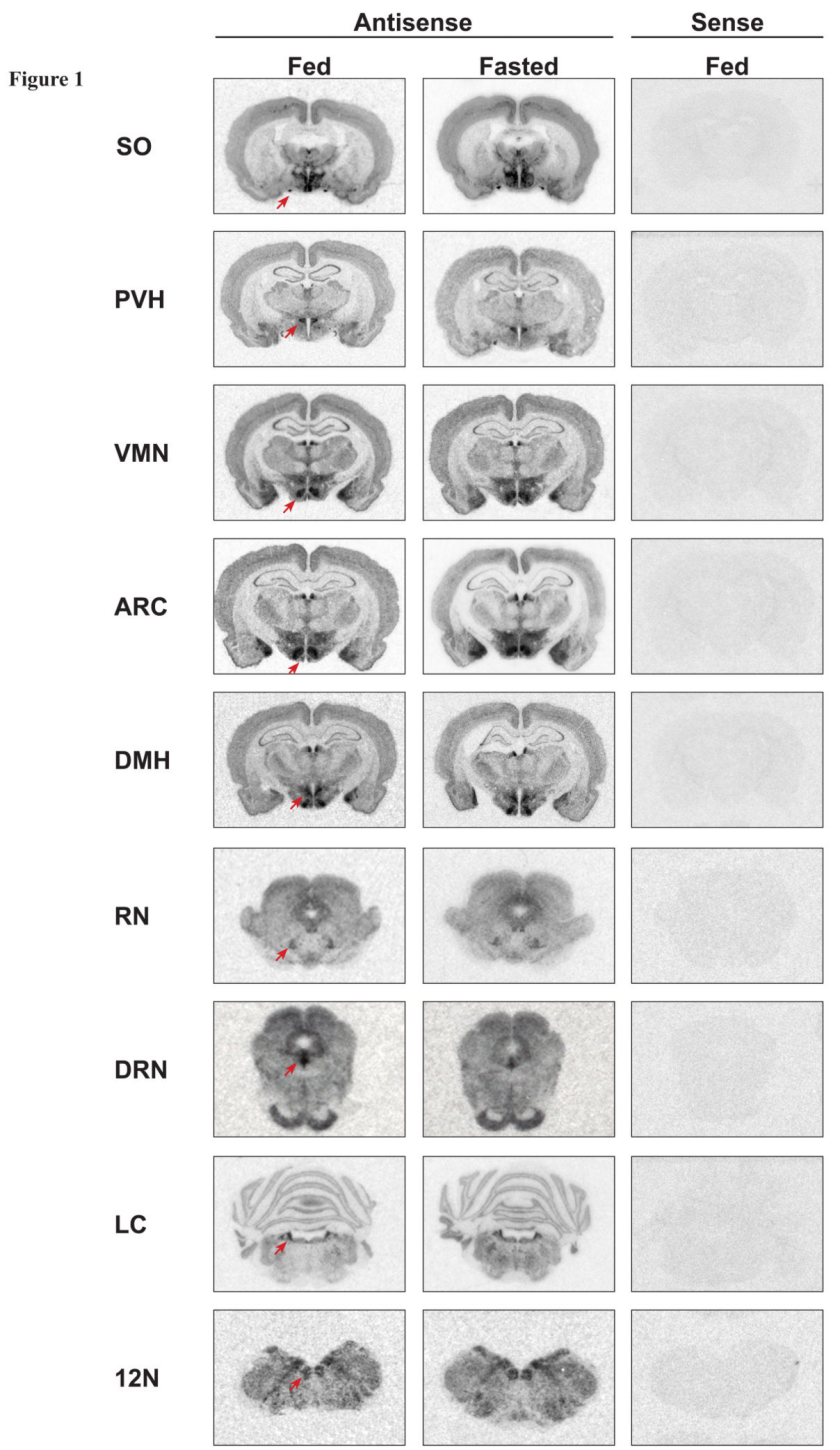
Representative expression of NOP receptor mRNA using ISHH in the SO, PVH, VMN, ARC, DMH, RN, DRN, LC and 12N of fed and fasted rats. Autoradiographs of representative coronal brain sections exhibiting hybridization of the [^35^S]-labeled riboprobes in brain tissue from fed and fasted rats. Red arrows indicate a positive hybridization signal within analyzed regions. Sense probe controls demonstrated an absence of hybridization, confirming the specificity of the antisense NOP receptor probe. Abbreviations: SO, supraoptic nucleus; PVH, paraventricular nucleus of the hypothalamus; VMN, ventromedial nucleus of the hypothalamus; ARC, arcuate nucleus of the hypothalamus; DMH, dorsomedial nucleus of the hypothalamus; RN, red nucleus; DRN, dorsal raphe nucleus; LC, locus coeruleus; 12N, hypoglossal nucleus.

**Fig. 2:**
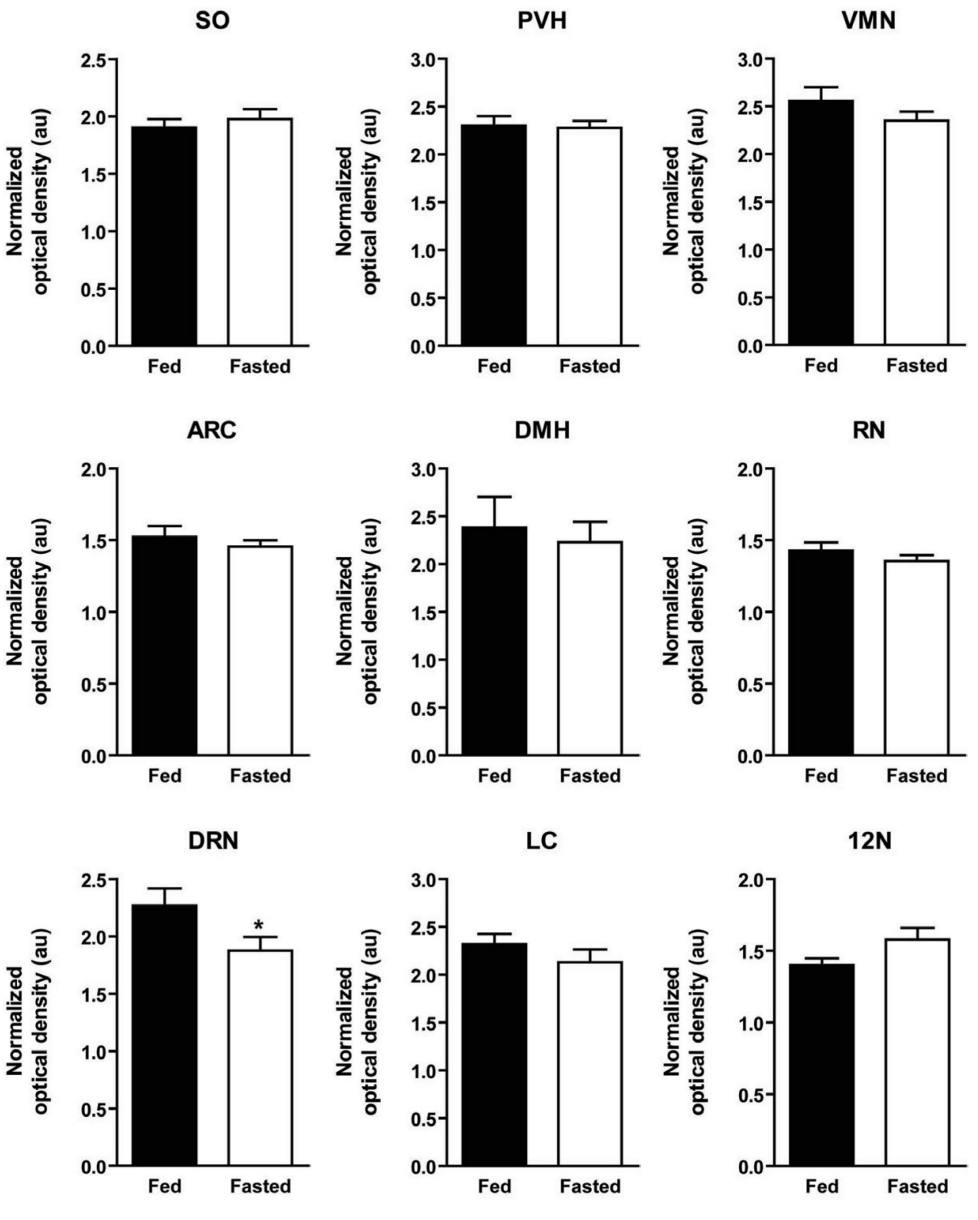
Quantification of NOP receptor mRNA expression in the SO, PVH, VMN, ARC, DMH, RN, DRN, LC and 12N from ISHH autoradiographs. The optical density of the positive hybridization signal within each analyzed region (as detected by the antisense NOP receptor riboprobe) was determined and compared in brain tissue from fed and fasted rats. Levels of NOP receptor expression were normalized to that of cortex. No differences in NOP receptor mRNA expression levels were detected in the SO, PVH, VMN, ARC, DMH, RN, LC or 12N. In the DRN, fasted rats exhibited a significant reduction in NOP receptor mRNA expression as compared to fed rats. Data are presented as the mean ± S.E.M. *, *p*<0.05. Abbreviations: SO, supraoptic nucleus; PVH, paraventricular nucleus of the hypothalamus; VMN, ventromedial nucleus of the hypothalamus; ARC, arcuate nucleus of the hypothalamus; DMH, dorsomedial nucleus of the hypothalamus; RN, red nucleus; DRN, dorsal raphe nucleus; LC, locus coeruleus; 12N, hypoglossal nucleus.
